
JOURNAL INFORMATION
==============================
NLM Title Abbreviation: Intensive Care Med Exp
Journal ID: Intensive Care Med Exp
NLM Journal ID: 101645149

Intensive Care Medicine Experimental

EISSN: 2197-425X

ARTICLE INFORMATION
==============================
PMCID: PMC7733698
PMID: 33313986
DOI: 10.1186/s40635-020-00354-8
Article ID: 354
Article version: 1
Subjects: Meeting Abstracts

ESICM LIVES 2020

Electronic publication date: 2020 Dec 14
Publication date: 2020 Dec
Volume: 8
Issue: Suppl 2
Issue sponsor: Publication of this supplement has not been supported by sponsorship.
Electronic Location ID: 73
Copyright: © The Author(s) 2020
License: Open AccessThis article is licensed under a Creative Commons Attribution 4.0 International License, which permits use, sharing, adaptation, distribution and reproduction in any medium or format, as long as you give appropriate credit to the original author(s) and the source, provide a link to the Creative Commons licence, and indicate if changes were made. The images or other third party material in this article are included in the article's Creative Commons licence, unless indicated otherwise in a credit line to the material. If material is not included in the article's Creative Commons licence and your intended use is not permitted by statutory regulation or exceeds the permitted use, you will need to obtain permission directly from the copyright holder. To view a copy of this licence, visit http://creativecommons.org/licenses/by/4.0/.
License URL: https://creativecommons.org/licenses/by/4.0/

Erratum in: Correction to: Dysregulated expression of the b2-adrenergic receptor insepsis 9 17 2 2021 7 Intensive Care Medicine Experimental PMC7889773 33595727
Erratum in: Correction to: ICU-outcomes in CAR-T patients—A single centre experience 9 18 2 2021 9 Intensive Care Medicine Experimental PMC7892649 33599849
Conference name: 33rd ESICM Annual Congress
Conference Acronym: ESICM LIVES 2020
Conference location: Virtual
Conference date: 06-09 December 2020
issue-copyright-statement: © The Author(s) 2020

==============================
Publisher's Note

Springer Nature remains neutral with regard to jurisdictional claims in published maps and institutional affiliations.

Change history

2/19/2021

A Correction to this paper has been published: 10.1186/s40635-021-00370-2

