# Correction to: Dysregulated expression of the b2-adrenergic receptor insepsis

**DOI:** 10.1186/s40635-021-00375-x

**Published:** 2021-02-17

**Authors:** A. Kleyman, A. Cesar, G. Stanzani, N. Arulkumaran, W. Khaliq, DT. Andreis, B. Bollen Pinto, M. Waugh, M. Singer

**Affiliations:** 1 University College London, Bloomsbury Institute of Intensive Care Medicine, London, UK; 2 Intensive Care Unit, University Hospital Lewisham, London, UK; 3 Anesthesiology, Hôpitaux Universitaires de Genève (HUG), Genève, Switzerland

## Correction to: Intensive Care Medicine Experimental 2020, 8(Suppl 2):73 10.1186/s40635-020-00354-8

After publication of this supplement [1], it was brought to our attention that the name of the third author of abstract 000607 had been misspelled: the author’s surname had been written as ‘Stanzami’ instead of ‘Stanzani’.

The name has now been corrected in the supplement and, furthermore, can be found in the author list of this correction.

For reference, please find the body of the abstract in question below:

**Introduction:** Catecholamine hypo-responsiveness is a hallmark of sepsis, but its pathogenesis remains elusive. The adrenergic pathway, from catecholamine binding to the adrenergic receptor (AR) to the final physiological effect is a multistep process. Knowledge about protein expression and localisation of different constituents of the adrenergic signalling in sepsis, including adrenergic receptors themselves, is sparse.

**Objectives:** Using our well-characterised 72 h fluid-resuscitated rat model of faecal peritonitis (1), where accurate prognostication can be made as early as 6 h, we analysed expression and membrane localisation of the b2-AR protein in heart tissue at early (6 h) and late (24 h) timepoints.

**Methods:** Sepsis was induced in male Wistar rats by i.p. injection of faecal slurry under general anaesthesia. The animals were then recovered and i.v. fluid resuscitation was started 2 h after sepsis induction. Sham-operated rats (SH) were treated identically but did not receive the slurry. At 6 h, an echo-measured heart rate cut-off of 460 bpm was used to classify animals into predicted survivors (SR) and non-survivors (NSR). Rats were culled at either 6 or 24 h with heart tissue collected and snap frozen in liquid nitrogen. To study b2-AR expression, either protein lysates of whole heart tissue or membrane fractions were prepared and analysed by Western Blot with antibodies against b2-AR and PFK or Caveolin 3. Specific signals were nalysed in Image Studio Lite 5.2. Results are presented as mean ± SE and considered significant at *p* < 0.05 (Student’s *t*-test).

**Results:** The level of expression of b2-AR myocardial protein expression did not change in survivors but declined in non-survivors being significantly lower than survivors at 24 h (Table 1). Representative results of membrane localisation of b2-AR within different membrane fractions are presented in Fig. 1. Lipid rafts were observed in only two of the 12 gradient fractions, namely fractions 4 and 5. This was confirmed by cholesterol distribution and caveolin 3 staining. In sham operated animals and in predicted survivors at 24 h, b2-AR appeared strongly in fraction 5, associated with the lipid rafts. In predicted non-survivors, b2-AR was not detected in fractions 4 and 5 either at 6 or 24 h post-sepsis.

**Conclusion:** More severe sepsis, with corresponding poor prognosis, is accompanied by increasing catecholamine hyporesponsiveness. One cause could be a decrease in adrenergic receptor protein expression. Localisation of the b2-AR within lipid rafts is also a prerequisite for their normal functioning. In predicted non-survivors myocardial b2-AR was dissociated from the lipid rafts. Further studies are necessary to identify strategies that restore adrenergic signalling in sepsis.
