# Correction to: ICU-outcomes in CAR-T patients—A single centre experience

**DOI:** 10.1186/s40635-021-00370-2

**Published:** 2021-02-18

**Authors:** T. Materski, M. John, T. Pirani, R. Benjamin, A. Kuhnl, V. Potter, R. Sanderson, V. Metaxa

**Affiliations:** 1 Critical Care, Kings College Hospital, London, UK; 2 Hematol-Ogy, Kings College Hospital, London, UK

## Correction to: Intensive Care Medicine Experimental 2020, 8:73 https://doi.org/10.1186/s40635-020-00354-8

After publication of this supplement [1], it was brought to our attention that the name of the first author of abstract 000954, Tomasz Materski, had been miswritten: the first and last name had been erroneously swapped.

The name has now been corrected in the supplement and, furthermore, can be found in the author list of this correction.

For reference, please find the body of the abstract in question below.

**Introduction:** Chimeric antigen receptor-modifed T (CAR-T) cells are a revolutionary treatment for patients with advanced blood cancers. A proportion of these patients will develop signifcant toxicities, requiring critical care management. Our hospital was one of 7 in the UK approved for the treatment of relapsed/refractory high-grade lymphoma and refractory acute lymphoblastic leukaemia (ALL).

**Objectives:** To describe our experience with lymphoma and ALL patients, who received CAR-T CD19 cells and were admitted to the intensive care unit (ICU).

**Methods:** From January 2019 until May 2020, patients with difuse large B cell lymphoma (DLBCL), transformed follicular lymphoma, primary mediastinal B-cell lymphoma (PMBCL) or ALL, who were infused with CAR-T CD19 cells and developed ≥ grade 2 Cytokine Release Syndrome (CRS) and Immune Efector Cell Associated Neurotoxicity Syndrome (ICANS) were analysed.

**Results:** Sixty-one patients underwent CAR-T therapy during the study period, with 21 (34%) requiring admission to ICU. The mean age at presentation to ICU was 57 years and 76% of the admissions were males. All patients were receiving broad spectrum antibiotics and 19% of treatments were broadened in ICU and an antifungal was added. CRS/sepsis (n = 12, 57%) was the most common documented reason for admission to ICU, with a proven infection documented in only 2 patients. The reasons for admission are shown in Fig. 1. Median duration of ICU stay was 4 (range 1–21) days. Grade 2 CRS was documented in 12 patients (57%) for a median duration of 2 (range 0–6) days. Grade 3 CRS was seen in 6 patients (29%) for 1–3 days. No patient progressed to grade 4 CRS during their ICU stay. Six patients had grade 1 ICANS each for a median duration of 4 (2–6) days, whereas another 6 had grade 3 ICANS for 2 days (1–4). During their ICU stay, 9 patients (48%) required vasopressors for a median duration of 2 (1–3) days. Three patients (14%) needed mechanical ventilation with a median duration of 5 (4–9) days and 2 patients needed renal replacement therapy for a median duration of 4 (1–7) days. IL-1, IL-6 antagonists and steroid administration are shown in Fig. 2. Three patients (14%) died in ICU, all in multi-organ failure. Two deaths were attributed to invasive pulmonary aspergillosis and the third to disease progression. Further 7 patients that were discharged alive from ICU, died before hospital discharge from disease progression.

**Conclusion:** A signifcant number of patients receiving CAR-T therapy will need admission to ICU, requiring various level of organ support. Diferentiation between CRS and neutropenic sepsis was difcult, and all patients stayed on antibiotics for the duration of their ICU stay. The observed ICU mortality (14%) was in accordance to the one reported in international literature and no deaths were attributed to treatment toxicity. Close collaboration between haematology and ICU is warranted for the optimal management of CAR-T patients.
